# Controversies about sugars consumption: state of the science

**DOI:** 10.1007/s00394-016-1227-8

**Published:** 2016-06-20

**Authors:** James M. Rippe, Ascensión Marcos

**Affiliations:** 1 Rippe Lifestyle Institute, 21 North Quinsigamond Avenue, Shrewsbury, MA 01545 USA; 2 Rippe Lifestyle Research Institute of Florida, 215 Celebration Place, Celebration, FL 34747 USA; 3 University of Central Florida, 4000 Central Florida Boulevard, Orlando, FL 32816 USA; 4 Department of Metabolism and Nutrition, Institute of Food Science, Technology and Nutrition (ICTAN), Spanish National Research Council (CSIC), C/José Antonio Novais, 10, 28040 Madrid, Spain

**Keywords:** Sucrose, High-fructose corn syrup, Isoglucose

## Abstract

Few topics in nutrition generate more controversy and debate than the putative associations between added sugars and health. With this as background, a group of researchers in the area of sugars and health gathered at the European Nutrition Conference (FENS) in 2015 to discuss these controversies and provide evidence-based science. The purpose of the current article was to provide a brief summary of some of the highlights from each of the presenters and serve as an Introduction to the supplement which contains full articles based on their presentations.

The consumption of added sugars and their putative effects on various conditions such as obesity [1–3], risk factors for coronary heart disease (CHD) [4–9], diabetes (T2D) [10–13] and metabolic syndrome continue to stimulate considerable controversy [14–18].

In addition to active debate within the academic and nutrition professional communities, numerous articles have been written in the popular press, often containing inflammatory headlines and frequently failing to accurately portray modern scientific understandings of the role of added sugars in human nutrition. Headlines such as “Pure, White and Deadly” [19] or “Death by Sugar” [20] or “Sugar, Drastic Measures” have frequently been written. The prestigious *New York Times* devoted a cover story to their magazine entitled “Sweet and Vicious” [21] related to sugars consumption and posed the question “Is Sugar Toxic?” Even prestigious medical journals such as the *British Medical Journal* have posted articles on its Web site with the inflammatory headline “Sugar is the New Tobacco” [22].

While it has been argued that added sugars consumption, in general, is associated with increased risk of various metabolic diseases, much of the focus has been on the fructose component of many sugars which is contained in roughly equal proportions to glucose in the two leading sources of added sugars in the human diet, namely sucrose and high-fructose corn syrup (HFCS) which is also called “isoglucose” in Europe [23].

Even though sugar (sucrose) has been part of the human diet for millennia, it did not attract much attention from nutrition critics until the 1970s. In 1972 and again updated in 1986 and 2012, John Yudkin’s book “Pure, White and Deadly: How sugars are killing us and what we are doing to stop it” was one of the first to suggest nutritional differences between simple sugars and complex carbohydrates [25]. Yudkin posed that sugars had adverse effects even when consumed at levels typical of the human diet.

Modern controversies about sugars appear to have started in the early 2000s. One article that garnered considerable attention was published in 2004, when Bray and Popkin wrote a commentary in the *American Journal of Clinical Nutrition* suggesting a temporal correlation between increased use of HFCSin the USA and rising rates of obesity [24]. Bray and Popkin posed the hypothesis that “consumption of HFCS in beverages may play a role in the epidemic of obesity.” Even though the authors cautioned that they were only raising a hypothesis, and that temporal associations do not establish cause and effect, it quickly was misinterpreted as fact by many members of the media, some internet commentators and even some physicians and scientists.

In the 1980s and 1990s, a series of scientific papers by Reavan and Reiser focused attention on the fructose component of sucrose and HFCS as posing particular problems related to heart disease and metabolic syndrome. Many of the arguments that were promulgated by these two investigators and others were addressed in a 1993 fructose monograph edited by Forbes and Bowman “Introduction to the health effects of dietary fructose,” which was published as a Supplement to the *American Journal of Clinical Nutrition* [26]. This monograph concluded “on the basis of currently available information there is little basis for recommending increased or decreased use of fructose in the general food supply or in products or special dietary use.”

A considerable amount of scientific research launched since 2004 has focused on potential relationships between fructose-containing sugars and the obesity epidemic and other related health problems. It should be noted that some portion of the research conducted in this time frame compared pure glucose to pure fructose [27, 28], neither of which is consumed in isolation in any appreciable degree in the human diet. Other research utilized fructose-containing sugars in amounts far greater than people can generally consume in real-world settings [16]. Numerous epidemiologic studies and review articles have also been published in this time frame suggesting a link between sugars consumption, particularly in the form of sugars-sweetened beverages (SSBs), and various adverse metabolic consequences [1, 3, 4, 29–32].

Recently, various scientific and health organizations have joined in this debate by recommending upper limits of sugars consumption far lower than typically consumed by individuals in the industrialized world. The World Health Organization (WHO) [33], Scientific Advisory Committee on Nutrition in England (SACN) [34] and the American Heart Association (AHA) [35] have all proposed dramatically reducing upper limits of sugars consumption to levels of 10 % of calories consumed, or less. The 2015 Dietary Guidelines Advisory Committee (2015 DGAC) [36, 37] also recommended the reduction of upper limit of no more than 10 % of calories from added sugars. This recommendation from the 2015 DGAC also formed the basis for the Food and Drug Administration (FDA) in the USA recommending an upper limit of added sugars consumption of no more than 10 % of calories and proposing a rule listing added sugars as a separate line item on the nutrition facts panel [38].

Many of these recommendations have been opposed by members of the scientific community as not encompassing the totality of available science, particularly from randomized controlled trials or prospective cohort studies. Furthermore, other scientific organizations such as the European Food Safety Administration (EFSA) [39] found no harm, and even some benefit, in fructose consumed up to 25 % of calories, while the Institute of Medicine Carbohydrate Report also found no harm in fructose-containing sugars when consumed at less than 25 % of calories [40] It is also important to emphasize that physical activity has an impact on the metabolic handling of fructose; thus, nutritional considerations should be put in the context of overall lifestyle habits and practices [41–43].

In this contentious environment, a group of experts in various aspects of metabolism convened at the 12th European Nutrition Conference, FENS 2015 in Berlin, Germany, to conduct a session entitled “Controversies about sugar consumption: state of the science.” The intent of this symposium was to summarize recent scientific evidence related to the relationship between sugars consumption and various metabolic, physiologic and neurologic sequelae. The articles contained in this supplement are based on presentations made at this conference.

The first paper in this supplement by Sievenpiper and colleagues presents results from systematic reviews and meta-analyses related to obesity and diabetes largely conducted within their research group. Sievenpiper started by exploring the hierarchy of evidence in research trials as outlined in guidelines from England and the USA [44] (see Fig. 1).

**Fig. 1:**
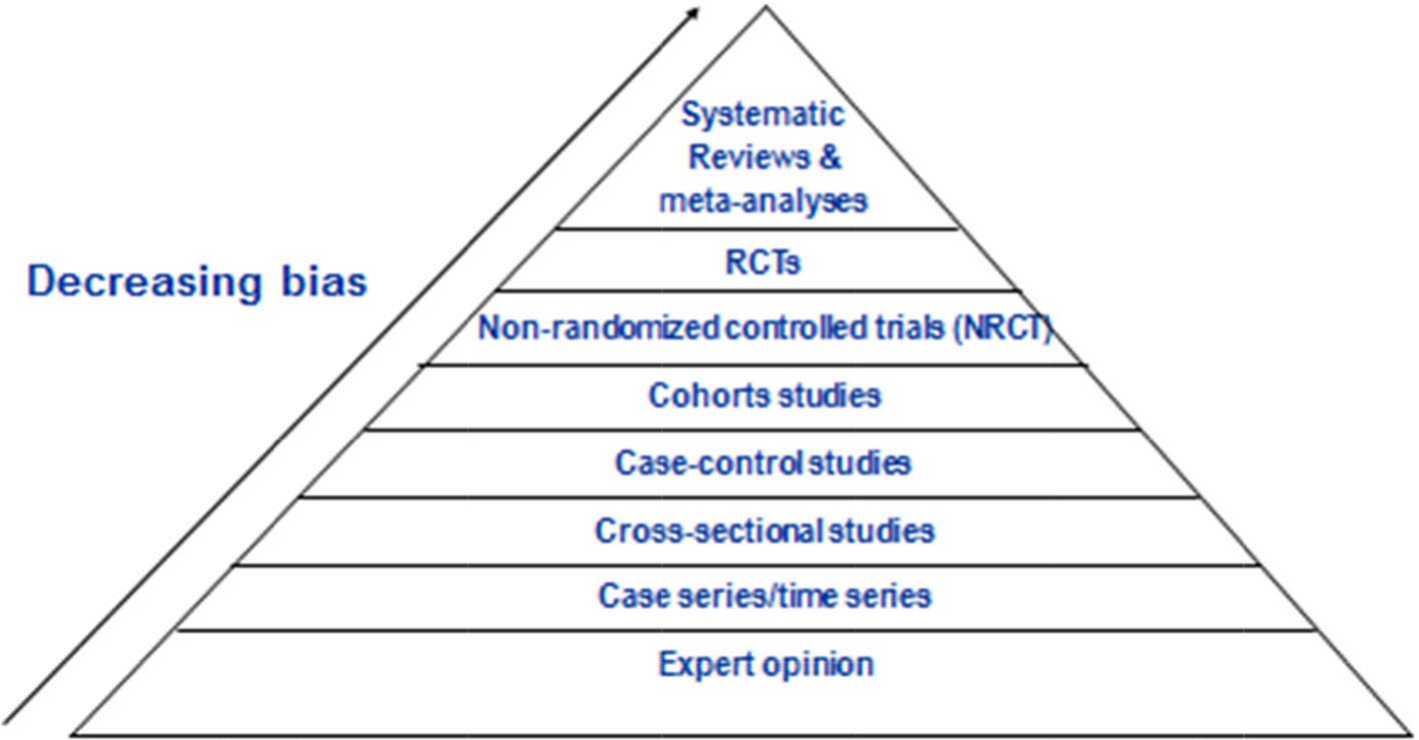
Hierarchy of evidence in evidence-based medicine. © Prof. Dr. John Sievenpiper, with permission

As depicted in this figure, the top half of the pyramid showing the least likelihood of bias includes cohort studies, non-randomized controlled trials (NRCT), randomized controlled trials (RCTs) and systematic reviews and meta-analyses. Sievenpiper et al. describe prospective cohort studies published by members of their research group or others involving sugars and weight change, diabetes risk, hypertension risk, gout risk and CHD risk. They conclude that there is little evidence suggesting that sugars consumption within the normal range of human consumption [in the USA, over 87 % of the population had estimated intakes of added sugars <25 % of total calories which equates to <92 grams (day)] [45] leads to increased risk of these metabolic consequences with the sole exception of gout risk. The authors suggest that SSBs may pose more risk than other sources of added sugars, but caution that the relationship is only seen in extreme quantiles analyses with few exceptions, the effect sizes are low (RR < 1.3) and the significance is greatly attenuated by adjustment for energy. Furthermore, residual confounding may exist since consumers of SSBs eat more calories, exercise less, smoke more and have a poor dietary pattern. The authors further remind us that many different foods may contribute to weight change and cite a paper by Mozaffarian et al. [46] which demonstrates that SSB consumption falls behind potato chips and potatoes (particularly French Fries) and is similar to processed and unprocessed red meat with regard to association with weight gains.

The authors provide a review of different types of controlled trials including “substitution” trials, “addition” trials and ad libitum trials with regard to added sugars. They note that the contribution of fructose-containing sugars is difficult to separate from other factors which contribute to the epidemic of obesity and cardiometabolic disease. Furthermore, they argue that any effect of sugars on these risk factors is highly dependent on energy balance and nutrient adequacy. They caution that attention needs to remain focused on reducing overconsumption of all caloric intake including those high in added sugars, whole promoting healthier dietary patterns and increasing physical activity.

Rippe and Angelopoulos in their article “Sugars, Non-nutritive Sweeteners, Obesity and Cardiovascular Disease” present data from RCTs utilizing various levels of HFCS, sucrose, fructose and glucose both from their research laboratory and those of others related to obesity and cardiovascular disease risk factors. These authors remind us that heart disease and stroke remain dominant causes of mortality in both the USA and Europe.

Their article begins with a presentation of theoretical concerns for why differences in metabolism of fructose and glucose in the liver could potentially lead to adverse metabolic effects. It has long been known that fructose and glucose are handled differently in the liver [47]. Over 90 % of ingested fructose is cleared on first pass and metabolized in the liver. A small amount of the fructose taken up by the liver may be converted in the process of de novo lipogenesis into fatty acids (on the order of 1–5 %). These fatty acids are converted to triglycerides in hepatocytes and are released into the systemic circulation complexed with the VLDL.

Several aspects of metabolism of fructose in the liver are important to emphasize. Multiple studies have demonstrated that approximately 50 % of the fructose is converted into glucose and a substantial portion of metabolized fructose appears to be directly stored as glycogen in the liver (approximately 15 %) [46, 47]. Additionally, about 25 % of fructose is converted into lactate. Thus, only a minor portion of the oral fructose is converted into fatty acids (on the order of 1–5 %) [47–49]. Although this is a minor pathway, it has been postulated by some investigators to potentially play a role in the development of fructose-induced hepatic steatosis, particularly when large doses of fructose are administered.

Rippe and Angelopoulos report that experiments conducted in their laboratory at dosage levels between the 25th and 90th percentile population consumption level of fructose have not shown any lipid abnormalities, with the sole exception of triglycerides which often rise when levels above 20 % of calories in added sugars are consumed. Rippe and Angelopoulos further report that there are no differences between sucrose and fructose with regard to energy-regulating hormones or appetite. They note that in the USA, an average increase of 474 calories per person has occurred between 1970 and 2010, but that only 7 % of this increase comes from all added sugars combined [50]. The authors also report no adverse effects on blood pressure from sugars consumed within the normal levels of human consumption in studies lasting up to 10 weeks and no increased risk of obesity, although a slight weight gain occurred at levels between 90 and 95 % population consumption. In addition, no adverse effect on risk factors for diabetes or the metabolic syndrome and no differences between HFCS, sucrose, fructose and glucose with regard to hypothalamic blood flow were found in these studies.

The article by MacDonald focuses on the relationship between sugars and insulin resistance and diabetes. MacDonald presents data that insulin resistance and blood glucose levels are related to a variety of other metabolic conditions including dyslipidemia, CHD, hypertension, hyperinsulinemia and T2D. MacDonald notes that the proposed linkages linking sugars consumption to diabetes provide a mixed picture. Some animal studies have suggested this linkage exists as have some econometric analyses [10, 11]. Animal studies, however, may not translate well into humans, and econometric studies are considered to be a weak form of evidence. As noted by the author, the epidemiologic literature in this area is mixed as is evidence from RCTs. Some studies have suggested that high levels of fructose consumption (between 210 and 280 g of fructose/day) may increase liver fat and produce hepatic insulin resistance. As noted by the author, a study by Johnston et al. [51] in 32 overweight men with central adiposity showed that when these individuals were in energy balance, fructose and glucose had no effect on liver fat content. With overfeeding, however, fructose and glucose both increased liver fat content.

MacDonald points out evidence reviewed in the SACN report [38] stating that studies provide “no consistent evidence of an association between diets differing in the proportion of sugars in relationship to the incidence of T2D.” MacDonald notes that fructose or sucrose consumption may affect insulin sensitivity only at high intakes (>100 g fructose/day) and that overeating is associated with increased liver and muscle fat, but that the effect is similar for fructose and glucose. There is some evidence of association between SSB consumption and diabetes risk. However, this evidence is confounded by the association between sugars and high energy intake.

The article by Westwater, Fletcher and Ziauddeen tackles the controversial subject of “sugar addiction.” The authors pose the question of whether sugar “addiction” truly exists, based on modern science. The authors start by asking whether or not sugar acts like drugs of addiction. The authors review animal data showing that in rat models there is an increased dopamine response to sucrose, but only when it is available intermittently. This increased dopamine response to sucrose also occurs even in sham-fed rats, animals that consume the sucrose solution orally, but have it removed immediately via an implanted gastric cannula. This suggests that it is the sweet taste of sucrose rather than its caloric content, which, in combination with the intermittent access, results in the increased dopamine response. They raise the question of whether or not “sugar addiction” exists in human beings or, in more general terms, is there such a thing as more generalized “food addiction.”

The authors argue that though foods and drugs may seem to act on the same reward system, the neuroscience of drug addiction appears to be quite different than response to food. Indeed, even the observation that foods and drugs act on the same reward circuits in the brain is not accurate as drugs are thought to hijack the same brain circuits that subserve food reward. They review the Yale Food Addiction Scale (YFAS) raising questions about its reliability and validity [52]. The authors note that dopamine receptor studies in obese individuals have yielded conflicting results as have functional magnetic resonance imaging (fMRI) studies attempting to find a common neural mechanism for obesity, let alone food addiction, in human beings. The authors conclude that while animal models offer proof of concept for the possibility of “sugar addiction,” there is little human evidence to support general “food addiction,” let alone “sugar addiction.” They go on to point out that utilizing the concept of “food addiction” in areas such as diagnosis and treatment, policy change or legislation, or as a mechanism for approaching the obesity crisis all offer severe limitations.

Scientific research, particularly in the area of nutrition, has changed dramatically over the past 10 years. We are in the midst of worldwide pandemics of non-communicable diseases such as obesity and diabetes. CHD remains the leading cause of mortality worldwide. Moreover, the large venue of non-refereed information provided by the internet, in general, and social media, in particular, creates different communications realities than most scientists are equipped to handle. Public policy and regulations are at risk for being based on emotion, rather than the highest level of scientific information available.

In the particularly controversial area of added sugars and health, it is important that members of the scientific community base their conclusions on the highest level of science available. We hope that the articles provided in this supplement will provide the scientific community with one such avenue of evidence. We also wish to emphasize as did all of the speakers at the symposium that nutritional issues such as consumption of added sugars need to be placed in the context of overall lifestyle decisions such as weight management and physical activity.

## Abbreviations

2015 DGAC: 2015 Dietary Guidelines Advisory Committee
AHA: American Heart Association
CHD: Coronary heart disease
EFSA: European Food Safety Administration
FDA: Food and Drug Administration
HFCS: High-fructose corn syrup
fMRI: Functional magnetic resonance imaging
NRCT: Non-randomized controlled trial
RCT: Randomized controlled trial
SACN: Scientific Advisory Committee on Nutrition in England
SSBs: Sugars-sweetened beverages
T2D: Type 2 diabetes
VLDL: Very low density lipoproteins
WHO: World Health Organization
YFAS: Yale Food Addiction Scale
